# Three Antibiotics Exert Differential Effects on the Larval Microbiome and Fitness of *Hyphantria cunea*

**DOI:** 10.3390/microorganisms13092078

**Published:** 2025-09-06

**Authors:** Tong-Pu Li, Zhi-Heng Wang, Chen-Hao Wang, Bing-Ren Hao, Si-Ying Song, Zhuoma Dawa, Han Lei, Lv-Quan Zhao

**Affiliations:** Co-Innovation Center for Sustainable Forestry in Southern China, College of Forestry and Grassland, Nanjing Forestry University, Nanjing 210037, China; tpli@njfu.edu.cn (T.-P.L.);

**Keywords:** *Hyphantria cunea*, antibiotics, microbiome, community structure, functional traits, fitness

## Abstract

The severe damage caused by the fall webworm *Hyphantria cunea* is closely related to its internal microbiota. However, due to the widespread use of antibiotics and their environmental persistence, the specific effects of various antibiotics on the microbiome and fitness of *H. cunea* larvae remain ambiguous. This study investigated the impacts of three antibiotics (tetracycline, rifampicin, and kanamycin) on microbiome assembly, functional traits, and host fitness. Our findings revealed that each antibiotic distinctly altered the microbial community: tetracycline primarily decreased bacterial diversity (e.g., reduced abundance of *Actinomycetota*) and suppressed host fecundity; kanamycin lowered microbial evenness (e.g., decreased *Bacillota*) and diminished pupal weight; whereas rifampicin significantly restructured the community (e.g., increased *Pseudomonas* and decreased *Bacillota*), enhanced functional traits such as biofilm formation and stress tolerance, and imposed multidimensional adverse effects on fitness (prolonged developmental duration, reduced pupal weight, and decreased hatching rate). Alterations in microbiome diversity, structure, and function were tightly correlated with the differential impacts of antibiotics on host fitness. This research elucidates the mechanisms by which antibiotics disrupt host–microbe interactions in *H. cunea*, offering a theoretical foundation for understanding antibiotic ecological repercussions and devising microbe-based green pest control strategies.

## 1. Introduction

Insects harbor highly diverse symbiotic microorganisms that form co-evolved symbiotic relationships with their hosts, profoundly influencing core life processes such as growth and development, nutrient metabolism, and immune defense [1,2,3,4,5]. For example, the cotton aphid *Aphis gossypii* relies on the obligate endosymbiont *Buchnera aphidicola* to synthesize essential amino acids (e.g., tryptophan) for normal development [6,7]. The symbiosis between *Aedes aegypti* and the endosymbiont *Wolbachia pipientis* significantly enhances its resistance to pathogens such as dengue virus and Zika virus [8,9]. *Pseudomonas fulva* in the gut of the silkworm *Bombyx mori* can efficiently degrade defensive secondary metabolites such as chlorogenic acid and rutin in mulberry leaves by secreting β-glucosidase, thereby helping the host break through plant chemical barriers [10]. The dynamic balance of microbial communities is also crucial for maintaining insect fitness [11]. Dysbiosis of the gut microbiota in *Drosophila melanogaster* and the honeybee *Apis mellifera* significantly reduces their respective survival rate and fecundity [12,13,14]. Moreover, the adaptability of the pear lace bug *Stephanitis nashi* to different host plants such as mulberry and paper mulberry relies more heavily on the dynamic adjustment of its gut microbiota [15]. Therefore, analyzing the assembly mechanisms and functional characteristics of insect microbial communities is an important basis for clarifying the adaptive evolution of pests and the mechanisms of their outbreaks.

The diversity and composition of insect microbial communities are dynamically regulated by biotic and abiotic factors. Biotic factors include insect species, developmental stages, and endosymbionts [16,17,18], while abiotic factors include environmental conditions such as antibiotics, temperature, and humidity [19,20,21]. Among them, antibiotics serve as key interfering factors, and their environmental residues can affect the microbial balance of non-target insects via food chain transfer, water cycling, and other pathways [21,22]. For example, agricultural and forestry residues of the broad-spectrum antibiotic tetracycline can disrupt the symbiotic relationship between *Snodgrassella* and *Gilliamella* in the honeybee gut microbiota, leading to increased colony mortality [13,23,24]. The combination of rifampicin and insecticides significantly reduces the weight and lifespan of aphids [25]. Kanamycin inhibits the growth of strains such as *Escherichia coli*, thereby leading to decreased fitness of *Plutella xylostella* larvae and ultimately population decline [26]. These cases indicate that antibiotic exposure may have significant negative impacts on insect fitness by disrupting nutrient absorption, impairing immune function (e.g., reducing antimicrobial peptide secretion), and decreasing reproductive efficiency [13,27]. Systematic evaluation of their ecological effects and mechanisms of action holds significant practical importance.

The fall webworm (*Hyphantria cunea*) is a globally invasive pest whose larvae exhibit voracious feeding habits and can rapidly consume leaves from over 300 plant species including poplar, willow, elm, and Metasequoia. This defoliation leads to loss of photosynthetic function and even death of trees, and also damages ecological barriers such as urban green spaces and farmland shelterbelts, inflicting severe ecological and economic harm to forestry [28,29,30]. Outbreaks of this pest are closely associated with the functional dynamics of its microbial community. Specific bacteria in the pest’s gut can significantly enhance the pest’s to new habitats by degrading plant secondary metabolites (e.g., terpenoids in *Metasequoia glyptostroboides*) [31,32]. In addition, *H. cunea* can acquire the bacterial-derived chitinase gene *HcuChiA* through horizontal gene transfer (HGT), thereby enhancing its intestinal antifungal immunity and thus breaking through the fungal barriers of host plants such as *M. glyptostroboides* [32,33]. However, environmental pressures such as pesticide use and antibiotic treatment can induce shifts in its microbiota structure, such disturbances may further impact its growth, development, and environmental adaptability [34,35]. Although studies have focused on the functions of the microbiota of *H. cunea*, the specific effects of tetracycline, rifampicin, and kanamycin on its fitness and microbiome remain unclear.

This study takes *H. cunea* as the research object to systematically explore the effects of tetracycline, rifampicin, and kanamycin on its fitness (such as developmental duration, pupal weight, and reproductive capacity) and microbiome (such as diversity, community structure, and functional phenotypes). The scope of this research is specifically restricted to these three tested antibiotics. By comparing key indicators between antibiotic-treated groups and control groups, the study focuses on characterizing their differential impacts on microbial community dynamics and host fitness. The objective is to clarify the variations in the regulatory mechanisms of host–microbe interactions mediated by different antibiotics and to provide empirical evidence for understanding the ecological consequences of antibiotic exposure for non-target insects.

## 2. Materials and Methods

This study focused on *H. cunea* to investigate the effects of different antibiotics (tetracycline, rifampicin, kanamycin) on its fitness and internal microbial community. The experiment was divided into three modules: insect rearing and sample collection, fitness determination, and microbiome sequencing and analysis. The specific methods are as follows:

### 2.1. Insect Rearing and Sample Collection

A total of 4 strains of *H. cunea* were used in the experiment, including 1 control strain (CK, reared on fresh mulberry leaves) and 3 treatment strains (TC, RFP, KAN, each reared on mulberry leaves soaked in 0.5% (*w*/*v*) tetracycline, rifampicin, and kanamycin solutions for 24 h, respectively). The original population was collected from Lianyungang City, Jiangsu Province, China in May 2024. The original population of the fall webworm (*H. cunea*) was collected in May 2024 from Lianyungang City, Jiangsu Province, China. Adults were identified based on the following characteristics: (1) Wing venation: The forewings have distinct radial cells, and the hind wings exhibit a fan-shaped venation. (2) Body coloration: The forewings are white to pale yellow with black spots; the male hind wings are orange-yellow, and the female hind wings are white. (3) Antenna type: The antennae are bipectinate in males and pectinate in females. Larvae were identified by the following features: (1) Setal arrangement: Each of the 1st to 7th abdominal segments has two longitudinal rows of setae, with 6 setae in the anterior row and 4 setae in the posterior row, and the bases of the setae have black spots. (2) Body color pattern: There are longitudinal white dorsal stripes, and lateral black warts are present, each bearing one black seta. (3) Leg structure: The fore tibiae have a pair of black terminal spines, while the mid and hind legs are reduced.

After collection, the population was maintained through continuous rearing on fresh mulberry leaves for 3 generations under laboratory conditions (26 °C, 16-h light/8-h dark photoperiod) to obtain the CK strain with a consistent genetic background. The establishment process of the treatment strains was as follows: fresh mulberry leaves were soaked in the corresponding 0.5% (*w*/*v*) antibiotic solutions (4 °C in the dark) for 24 h, after which they were removed. Excess liquid on the leaf surface was gently blotted off with sterile filter paper, and air-dried at room temperature for subsequent use; subsequently, newly hatched first-instar larvae were fed with the pre-treated mulberry leaves until pupation to establish TC, RFP, and KAN strains respectively. An antibiotic concentration of 0.5% (*w*/*v*) was a commonly used effective concentration in insect symbiotic microorganism interference experiments. Studies on the diamondback moth (*Plutella xylostella*) and aphids have confirmed that this concentration can effectively disrupt the hosts’ intestinal microbial community while avoiding acute larval death, allowing for the complete observation of life-history processes such as pupation and eclosion [25,26]. Additionally, unifying the soaking duration (24 h) with this concentration further ensured the consistency of comparison among the TC, RFP, and KAN groups. Meanwhile, this concentration simulated the potential residual levels of antibiotics in agricultural environments (farmland residues are usually in the range of 0.1–1.0%, *w*/*v*), which was consistent with the field exposure scenario of *H. cunea*, thereby enhancing the ecological value of the results [21]. During the rearing period of all strains, fresh mulberry leaves were replaced daily to ensure food supply and the effectiveness of antibiotic treatment. During sample collection, 5 fourth-instar larvae from each strain were pooled to form one sample pool (4 sample pools per strain). The samples were soaked in 75% ethanol for 90 s for disinfection, rinsed three times with sterile deionized water to remove surface microorganisms, and then immediately stored at −80 °C for subsequent genomic DNA extraction and high-throughput sequencing.

### 2.2. Insect Fitness Determination

To evaluate the effect of antibiotic treatment on the fitness of *H. cunea*, the pest’s biological characteristics (developmental duration, pupal weight) and reproductive indicators (fecundity and hatching rate) were measured. In the determination of developmental duration, four population boxes were established (20 newly hatched larvae per box). The duration of each instar (egg, first to sixth instar larvae), pupal stage, and adult stage were observed and recorded daily. One-way analysis of variance (One-way ANOVA) was used to test the significance of differences in the duration of each stage among the four strains (*p* < 0.05). For pupal weight determination, 30 female pupae and 30 male pupae were randomly selected from each strain, weighed accurately, and their weights recorded using an electronic balance (precision 0.0001 g). In the determination of reproductive indicators, twenty population boxes were established for each strain (2 male pupae + 1 female pupa per box). After eclosion, natural mating and oviposition were allowed, and the fecundity of each female and the egg hatching rate [(number of hatched larvae/total number of eggs) × 100%] were observed and recorded.

### 2.3. Microbiome Sequencing and Data Assembly

To clarify the effect of antibiotics on the microbial community of *H. cunea*, *16S rRNA* gene sequencing technology was used to analyze the bacterial community structure in its body. Genomic DNA of samples was extracted using the Qiagen DNeasy^®^ Blood & Tissue Kit (QIAGEN, Hilden, Germany). The V3–V4 hypervariable regions of the bacterial *16S rRNA* gene were targeted for PCR amplification using specific primers (forward primer: 5′-CCTAYGGGRBGCASCAG-3′; reverse primer: 5′-GGACTACNNGGGTATCTAAT-3′) were used for PCR amplification [36]. The PCR products were separated by 2% agarose gel electrophoresis and purified using AxyPrep DNA Gel Extraction Kit (Axygen, Union, CA, USA). After quantification with Qubit 3.0 (Thermo Fisher Scientific, Waltham, MA, USA), a PE250 (2 × 250 bp paired-end) sequencing library was constructed, and high-throughput sequencing was finally performed on the Illumina NovaSeq 6000 platform (Illumina, San Diego, CA, USA). The original sequencing data were demultiplexed based on index sequences and saved in FASTQ format. USEARCH (v10, drive5, Santa Cruz, CA, USA) was used to remove low-quality sequences (length < 200 bp, average quality score < 20) and chimeras. Operational Taxonomic Units (OTUs) were generated by clustering sequences at 97% similarity, and singleton OTUs were removed to reduce noise. The optimized OTU representative sequences were aligned against the Silva database (v138.1, ARB Team, Ludwig-Maximilians-Universität München, Munich, Germany) to generate an OTU table. For subsequent microbial community analysis and visualization, QIIME 2 (v2023.9, QIIME 2 Development Team, University of Colorado Boulder, Boulder, CO, USA; core function: comprehensive microbial community data analysis, including beta diversity calculation and PCoA analysis) and Circos toolbox (v0.69.9, Martin Krzywinski, Canada’s Michael Smith Genome Sciences Centre, Vancouver, BC, Canada; core function: generating circular visualization diagrams for microbial community structure and taxonomic correlation display) were used.

### 2.4. Microbial Diversity and Community Structure Analysis

To comprehensively evaluate the characteristics of the microbial community, uninformative and low-quality OTUs were first filtered out (screening criteria: OTUs were retained only if they contained at least 5 sequences in 3 samples and a total of at least 20 sequences across all samples). Alpha diversity analysis characterized the diversity of bacterial communities in each strain by calculating species richness (observed OTU number, Chao1 index, ACE index) and evenness (Shannon index, Simpson index) [25]. Of these indices, Chao1 and Shannon indices were used for visual display. Beta diversity analysis used permutational multivariate analysis of variance (PERMANOVA) to test the differences in microbial communities among different strains, and principal coordinate analysis (PCoA) was used for visualization based on Bray–Curtis distances. In addition, based on the relative abundance of OTUs at the phylum and genus levels, bacterial community structure plots were generated for each sample (genera with relative abundance < 1% were merged into “others” to simplify the display), and the corresponding relationship between each strain sample and microorganisms at the phylum/genus level was intuitively visualized using Circos diagrams.

### 2.5. Microbial Community Difference Analysis

To clarify the specific effects of different antibiotic treatments on the microbial community, Venn diagrams were used to quantify shared and unique bacterial taxa (phylum and genus levels) among the four strains, thus revealing the differences in community composition. Meanwhile, Student’s *t*-test was used to compare the differences in the relative abundance of microorganisms at the phylum/genus level between CK and each treatment group (with significance defined as *p* < 0.05) to identify microbial taxa with significant differences in abundance.

### 2.6. Microbial Function Prediction

To explore the effect of antibiotic treatment on microbial functions, BugBase (v0.1.0, Center for Computational and Genomic Medicine, Children’s Hospital of Philadelphia, Philadelphia, PA, USA) was used to predict the functions of the microbial community and identify significantly different microbial high-level phenotypes. The specific steps were as follows: first, the species abundance table and metadata were integrated into a BugBase-compatible biom format file; then, the default BugBase workflow was executed to predict metabolic pathways based on the KEGG Orthology (KO) database; outputs included phenotypic characteristics and the abundance data of corresponding functional pathways.

### 2.7. Statistical Analysis

Statistical analyses were conducted as follows: One-way analysis of variance (one-way ANOVA) was used to test differences in developmental duration among the four strains. Student’s *t*-test was applied to compare pupal weight, fecundity, egg hatch rate, phylum/genus-level microbial abundance, and functional pathway abundance between the control (CK) and each treatment group. Permutational multivariate analysis of variance (PERMANOVA), based on Bray–Curtis distances, was employed to assess differences in β-diversity in microbial communities. All statistical analyses were performed using R software (v4.3.2, R Foundation for Statistical Computing, Vienna, Austria): the ‘stats’ package was used for one-way ANOVA and Student’s *t*-test, and the ‘vegan’ package was used for PERMANOVA. For result visualization and supplementary statistical verification to ensure outcome consistency, GraphPad Prism (v9.5.1, GraphPad Software, San Diego, CA, US) was additionally used when necessary.

## 3. Results

To investigate the effects of different antibiotics on the fitness and microbiome of *H. cunea*, this study systematically analyzed the biological characteristics, microbial diversity, community structure, and functional changes of the control *H. cunea* strain (CK, reared on standard mulberry leaves) and three antibiotic-treated *H. cunea* strains (TC: tetracycline-treated, RFP: rifampicin-treated, KAN: kanamycin-treated).

### 3.1. Effects of Antibiotics on Fitness

**Figure 1:**
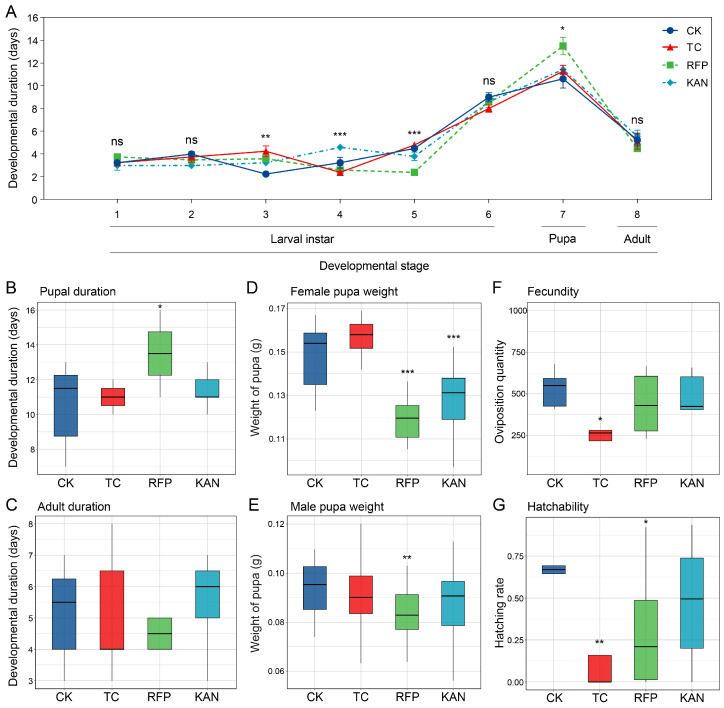
Effects of three different antibiotics on the fitness of *H. cunea*. (**A**) Developmental duration curves for the larval, pupal, and adult stages of the four *H. cunea* strains. Asterisks denote statistically significant differences in developmental durations across all stages among the four strains. (**B**,**C**) Comparisons of pupal developmental duration (**B**) and adult developmental duration (**C**) among the four *H. cunea* strains. (**D**,**E**) Comparisons of female pupal weight (**D**) and male pupal weight (**E**) among the four *H. cunea* strains. (**F**,**G**) Comparisons of fecundity (**F**) and hatchability (**G**) among the four *H. cunea* strains. Asterisks denote statistically significant differences between the control group (CK) and the antibiotic-treated groups (TC, RFP, or KAN) (*, *p* < 0.05; **, *p* < 0.01; ***, *p* < 0.001; ns, not significant). CK, control strain of *H. cunea* fed standard mulberry leaves; TC, *H. cunea* strain fed tetracycline-treated mulberry leaves; RFP, *H. cunea* strain fed rifampicin-treated mulberry leaves; KAN, *H. cunea* strain fed kanamycin-treated mulberry leaves.

All three antibiotics significantly reduced the fitness of *H. cunea*, but their target effects varied. In terms of developmental duration: at the 3rd instar larval stage, larval duration was significantly prolonged in the TC, RFP, and KAN groups (TC vs. CK: *t* = −3.703, *df* = 6, *p* < 0.01; RFP vs. CK: *t* = −3.813, *df* = 7, *p* < 0.01; KAN vs. CK: *t* = −2.828, *df* = 6, *p* < 0.05); at the 4th instar larval stage, only the KAN group showed a prolonged duration (*t* = −2.679, *df* = 7, *p* < 0.05); at the 5th instar larval stage, the effect was reversed in the TC group, with a significant shortening (*t* = 5.584, *df* = 7, *p* < 0.001); at the pupal stage, only the RFP group had a significantly prolonged duration (*t* = −2.478, *df* = 12, *p* < 0.05), while no significant differences were observed in adult lifespan (Figure 1A–C). Regarding pupal weight: the female pupal weight was significantly reduced in the RFP and KAN groups (RFP: *t* = 5.914, *df* = 30, *p* < 0.001; KAN: *t* = 4.113, *df* = 38, *p* < 0.001), and rifampicin also significantly reduced the male pupal weight (*t* = 3.195, *df* = 45, *p* < 0.01), with no significant effect observed in the TC group (Figure 1D,E). For reproductive indices: tetracycline significantly decreased fecundity (*t* = 2.558, *df* = 8, *p* < 0.05) and hatch rate (*t* = 4.444, *df* = 8, *p* < 0.01); rifampicin significantly reduced hatch rate (*t* = 2.497, *df* = 13, *p* < 0.05) (Figure 1F,G). In summary, tetracycline mainly inhibited reproductive capacity, kanamycin significantly reduced pupal weight, and rifampicin had negative impacts on developmental duration, pupal weight, and hatching rate.

### 3.2. Overview of Larval Microbiome

Through *16S rRNA* gene sequencing analysis, a total of 486 operational taxonomic units (OTUs) were identified larval intestinal microbiome across the four strains (CK, TC, RFP, KAN), belonging to 14 phyla, 23 classes, 50 orders, 97 families, and 165 genera (Supplementary Dataset S1). The sequencing quality was high, with a mean Good’s coverage of 99.99% (all samples ≥ 99.98%), indicating high data reliability (Table 1).

### 3.3. Effects of Antibiotics on Microbial Diversity

**Figure 2:**
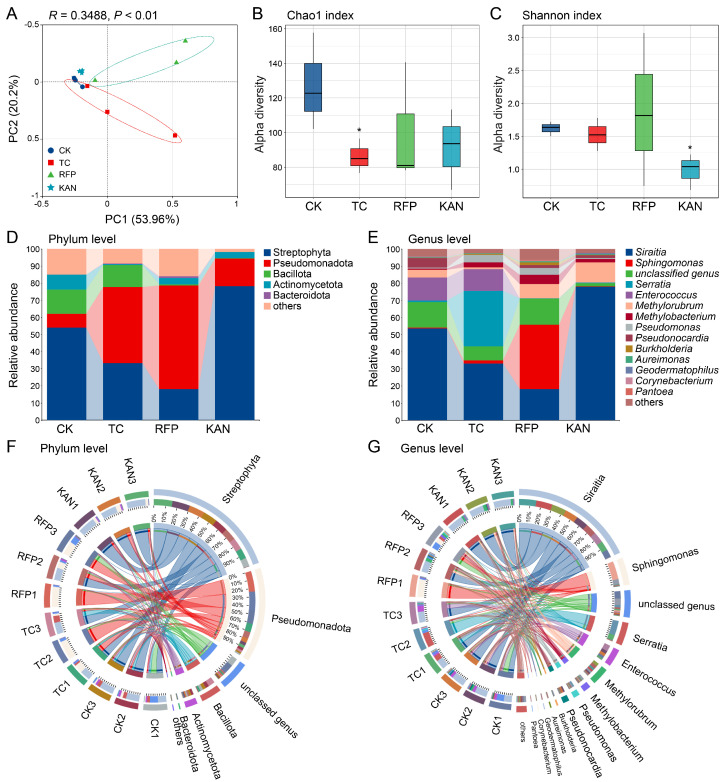
Effects of three different antibiotics on the diversity and structural composition of bacterial communities in *H. cunea*. (**A**) Principal coordinate analysis (PCoA) showing beta diversity patterns among the four *H. cunea* strains. (**B**,**C**) Alpha diversity metrics, including bacterial community richness (Chao1 index, (**B**)) and species evenness (Shannon index, (**C**)), across the four *H. cunea* strains. Asterisks denote statistically significant differences between the control group (CK) and the antibiotic-treated groups (TC, RFP, or KAN) (*, *p* < 0.05). (**D**,**E**) Structural composition of bacterial communities at the phyla (**D**) and genera (**E**) ranks, displaying predominant phyla and genera, with remaining taxa (relative abundance <1%) grouped as “others”. (**F**,**G**) Circos plots illustrating one-to-one correspondence between grouped samples and bacterial abundances, with remaining taxa (relative abundance <1%) grouped as “others”. Sample abbreviations are as in Figure 1.

Principal Coordinate Analysis (PCoA) showed that the first two principal coordinates (PC1 and PC2) together explained 74.16% of variation in microbial diversity (PC1: 53.96%, PC2: 20.2%). PERMANOVA analysis revealed a significant separation of microbial communities among different treatment groups (*R*^2^ = 0.3488, *p* < 0.01), suggesting that antibiotics significantly affected microbial diversity (Figure 2A). Further validation via alpha diversity analysis showed that the Chao1 index (species richness) in the TC group was significantly lower than that in the CK group (*t* = 2.41, *df* = 4, *p* < 0.05); the Shannon index (species evenness) in the KAN group was significantly lower (*t* = 3.749, *df* = 4, *p* < 0.05); while no significant change was observed in the RFP group (Figure 2B,C). These results indicated that tetracycline mainly reduced bacterial species richness, kanamycin decreased community evenness, and rifampicin had no significant effect on alpha diversity.

### 3.4. Effects of Antibiotics on Microbial Community Structure

**Figure 3:**
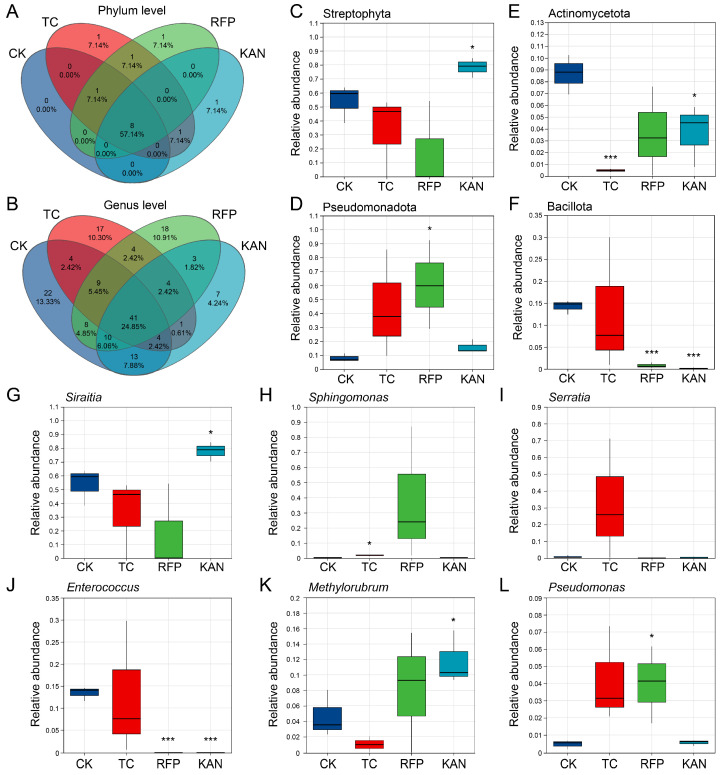
Comparative analysis of bacterial community differentiation among four *H. cunea* strains. (**A**,**B**) Venn diagrams visualizing shared and unique amplicon sequence variants (ASVs) for phyla (**A**) and genera (**B**), respectively. (**C**–**F**) Comparisons of relative abundances of key bacterial phyla: Streptophyta (**C**), Pseudomonadota (**D**), Actinomycetota (**E**), Bacillota (**F**). (**G**–**L**) Comparisons of relative abundances of key bacterial genera: *Siraitia* (**G**), *Sphingomonas* (**H**), *Serratia* (**I**), *Enterococcus* (**J**), *Methylorubrum* (**K**), *Pseudomonas* (**L**). Asterisks denote statistically significant differences between the control group (CK) and the antibiotic-treated groups (TC, RFP, or KAN) (*, *p* < 0.05; ***, *p* < 0.001). Sample abbreviations are as in Figure 1.

The main bacterial taxa at both phylum and genus levels were similar among the four strains, but significant differences existed in their relative abundances (Figure 2D–G). At the phylum level, the dominant phyla included Streptophyta, Pseudomonadota, Bacillota, Actinomycetota, etc.: the relative abundance of Actinomycetota in the TC group was significantly reduced (*t* = 8.452, *df* = 4, *p* < 0.001); in the RFP group, Pseudomonadota was significantly increased (*t* = −2.846, *df* = 4, *p* < 0.05) and Bacillota was significantly decreased (*t* = 13.744, *df* = 4, *p* < 0.001); in the KAN group, Streptophyta was significantly increased (*t* = −2.763, *df* = 4, *p* < 0.05), while both Actinomycetota (*t* = 2.746, *df* = 4, *p* < 0.05) and Bacillota (*t* = 15.408, *df* = 4, *p* < 0.001) were significantly decreased (Figure 3C–F). At the genus level, the dominant genera included *Siraitia*, *Sphingomonas*, *Serratia*, *Enterococcus*, *Methylorubrum*, etc.: *Sphingomonas* in the TC group was significantly reduced (*t* = −3.879, *df* = 4, *p* < 0.05); in the RFP group, *Enterococcus* was significantly decreased (*t* = 14.716, *df* = 4, *p* < 0.001) and *Pseudomonas* was significantly increased (*t* = −2.705, *df* = 4, *p* < 0.05); in the KAN group, *Siraitia* and *Methylorubrum* were significantly increased (*t* = −2.743, *df* = 4, *p* < 0.05; *t* = −2.701, *df* = 4, *p* < 0.05), and *Enterococcus* was significantly decreased (*t* = 14.716, *df* = 4, *p* < 0.001) (Figure 3G–L).

Venn analysis showed that at the phylum level, the shared bacterial phyla among the four strains accounted for 57.14% (8/14), while the proportion of phyla shared by 2–3 strains was relatively low; each of the three antibiotic-treated groups contained 1 unique bacterial phylum (1/14, accounting for 7.14%), whereas no unique phylum was found in the CK group (Figure 3A). At the genus level, the shared bacterial genera among the four strains accounted for 24.85% (41/165); the CK group had the most unique genera (22/165, accounting for 13.33%), followed by RFP (18/165, 10.91%), TC (17/165, 10.3%), and KAN (7/165, 4.24%), with the KAN group having the least (Figure 3B). These results indicated that antibiotics primarily altered microbial community structure by modifying the relative abundance of bacterial taxa rather than completely reshaping the community.

### 3.5. Effects of Antibiotics on Microbial Functions

Functional phenotypes refer to key biological traits of microbial communities, including the following: aerobic (requiring oxygen for respiration); anaerobic (surviving without oxygen); containing mobile elements (harboring genetic elements mediating horizontal gene transfer); facultatively anaerobic (adapting to both aerobic and anaerobic environments); forming biofilms (aggregating in self-secreted extracellular matrices); Gram-negative (having outer membrane and lipopolysaccharides in cell walls); Gram-positive (possessing thick peptidoglycan cell walls retaining purple Gram stain); potentially pathogenic (having disease-causing potential); and stress-tolerant (withstanding environmental pressures like toxins).

**Figure 4:**
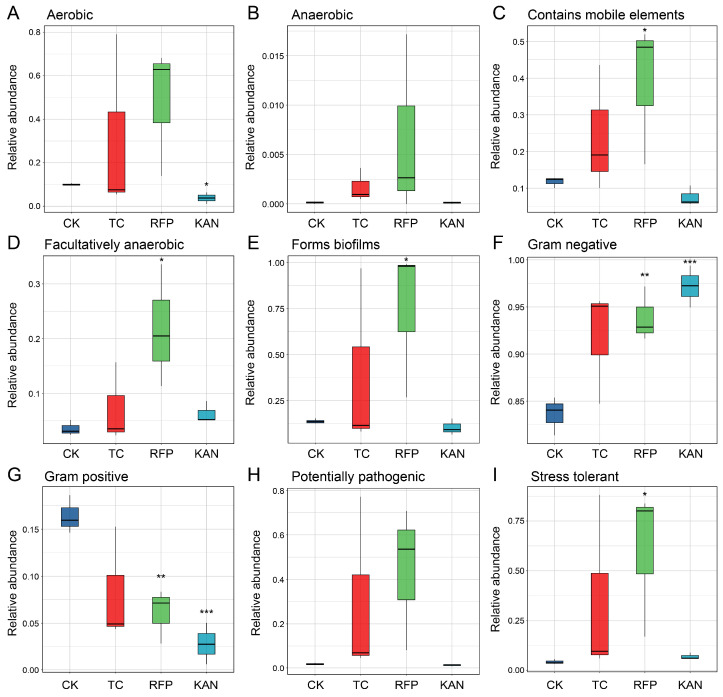
Comparative analysis of functional differences in bacterial communities among four *H. cunea* strains. To evaluate functional disparities across the four strains, BugBase-predicted high-level phenotypes were analyzed. The investigation compared nine phenotypic traits: (**A**) aerobic, (**B**) anaerobic, (**C**) contains mobile genetic elements, (**D**) facultatively anaerobic, (**E**) biofilm-forming capacity, (**F**) Gram-negative, (**G**) Gram-positive classification, (**H**) potential pathogenicity, and (**I**) stress tolerance. Different letters denote significant differences in pairwise comparisons. Asterisks denote statistically significant differences between the control group (CK) and the antibiotic-treated group (TC, RFP or KAN) (*, *p* < 0.05; **, *p* < 0.01; ***, *p* < 0.001). Sample abbreviations are as in Figure 1.

Functional prediction analyses revealed significant differences in these nine high-level phenotypes of the microbial communities among the four strains (Figure 4). No significant changes were observed in the functional characteristics of the TC group; in the RFP group, the functional abundances of mobile-element-containing, facultatively anaerobic, biofilm-forming, Gram-negative, and stress-tolerant traits were significantly increased (*t* = −2.413 to −5.033, *df* = 4, *p* < 0.05), while the functional abundance of Gram-positive traits was significantly decreased (*t* = 5.033, *df* = 4, *p* < 0.01); in the KAN group, the functional abundances of aerobic and Gram-positive traits were significantly decreased (*t* = 4.01, 7.845, *df* = 4, *p* < 0.05), and the functional abundance of Gram-negative traits was significantly increased (*t* = 7.845, *df* = 4, *p* < 0.001). In conclusion, rifampicin exerted the most pronounced effects on microbial functions, primarily exhibiting stimulatory effects; kanamycin exhibited both promoting effects on certain functions and inhibitory effects on others; and tetracycline showed the minimal impact.

## 4. Discussion

The complex interactions between insects, their microbial symbionts, antibiotics, and other environmental factors profoundly influence the balance of ecological systems [37,38,39,40]. As a globally invasive pest, the fall webworm *H. cunea* poses a severe threat to forest ecosystems and urban greenery [28,29,30]. This study systematically investigated the effects of three antibiotics (tetracycline, rifampicin, and kanamycin) on the microbiome assembly, fitness, and functional traits of *H. cunea* larvae. We found that different antibiotics reduced host microbial diversity, altered community structure and function to varying degrees, and ultimately inhibited host fitness. These results provide a theoretical basis for developing green pest control strategies based on microbial regulation.

### 4.1. The Differential Effects of Antibiotics on the Fitness of H. cunea

All three antibiotics significantly reduced the fitness of *H. cunea*, but their effects were specific: tetracycline primarily inhibited reproductive capacity (fecundity), kanamycin decreased pupal weight, and rifampicin exerted multidimensional negative impacts on developmental duration, pupal weight, and hatch rate. These differential phenotypes were closely related to the specificity of antibiotic action and their targeted interference with key microbial functions in host physiology [17,38,41,42]. The inhibitory effect of tetracycline on fecundity is consistent with previous studies showing that broad-spectrum antibiotics disrupt reproductive symbionts in insects [19,43]. For example, tetracycline reduces tick fecundity by altering nutrient-providing symbionts [44]. A similar mechanism may apply to *H. cunea*: the loss of symbionts involved in amino acid or vitamin synthesis (e.g., Actinomycetota with reduced abundance in the TC group) could impair oocyte development or egg viability [13,27,45]. The decrease in pupal weight caused by kanamycin may reflect disrupted nutrient metabolism: pupal weight was a key indicator of larval nutrient accumulation, and the reduced microbial evenness induced by kanamycin may destabilize the balance of nutrient-metabolizing taxa [44,45]. For instance, the reduction in Bacillota (rich in cellulolytic and protease-producing bacteria) in the KAN group may decrease the efficiency of decomposing plant secondary metabolites or complex carbohydrates in mulberry leaves, limiting energy intake for pupal development [26]. The extensive impairment of fitness by rifampicin (delayed development, reduced pupal weight, and decreased hatching rate) corresponds to its profound perturbation of microbial community structure and function: rifampicin increased the abundance of *Pseudomonas* (Gram-negative bacteria) while reducing Bacillota (Gram-positive bacteria), and enhanced traits such as biofilm formation and stress tolerance. These changes may disrupt symbiotic interactions—Bacillota (e.g., *Enterococcus*, whose abundance decreased in the RFP group) typically participate in nutrient absorption and immune regulation, whereas the overgrowth of *Pseudomonas* may alter intestinal pH or compete with beneficial symbionts, collectively inhibiting larval growth and reproduction [23,46].

### 4.2. Antibiotics Remodel Microbial Diversity and Community Structure

Antibiotics significantly altered the diversity and structure of the microbial community in *H. cunea*. Similar phenomena have been observed in other insects. For example, antibiotics have been shown to modify microbiome assembly and host fecundity in spider mites [19,32]; microbial community changes have been reported in tetracycline-treated fruit flies and honeybees [47,48,49]. The three antibiotics differed in their effects on microbial communities, reflecting their specific modes of action. Tetracycline reduced species richness by inhibiting bacterial protein synthesis, potentially eliminating a large number of sensitive taxa (e.g., *Sphingomonas* at the genus level), consistent with its broad-spectrum activity [45]. Kanamycin primarily decreased community evenness, suggesting selective inhibition of dominant taxa (e.g., Bacillota) and proliferation of less resistant groups (e.g., Streptophyta), thereby disrupting community balance; this unevenness may reduce functional redundancy [10,12]. Rifampicin did not alter alpha diversity but significantly shifted community structure, consistent with its mechanism of action (i.e., greater targeting of Gram-positive than Gram-negative bacteria) explaining the shift from Bacillota (Gram-positive) to *Pseudomonas* (Gram-negative) in the RFP group. Notably, all antibiotics primarily altered taxon abundance rather than eliminating core community members, indicating that the microbiome retains a core structure under perturbation, likely due to host selection for essential symbionts [15,50].

### 4.3. Effects of Antibiotics on Microbiome Function

Reduced microbial diversity implies simplified ecosystem functions, as different microorganisms typically play complementary and critical roles in nutrient cycling, disease resistance, and other processes [51,52]. Functional predictions revealed that antibiotic-induced microbial changes altered key traits, with rifampicin exerting the strongest impact, followed by kanamycin and tetracycline. These functional shifts provide mechanistic explanations for differences in host fitness. Rifampicin enhanced “contains mobile genetic elements” and “potential pathogenicity” traits, suggesting increased horizontal gene transfer (HGT) of antibiotic resistance genes or virulence factors. This could further disrupt host–microbe harmony—HGT of resistance genes may reduce the efficacy of future antibiotic exposure, while pathogenic taxa could impair intestinal integrity [53,54,55]. Additionally, enhanced “biofilm-forming capacity” and “stress tolerance” in the RFP group may reflect microbial adaptation to antibiotic pressure, but biofilms could hinder nutrient absorption by covering intestinal epithelium [46,56]. Kanamycin reduced “aerobic” and “Gram-negative” traits, consistent with its depletion of aerobic, Gram-positive Bacillota; aerobic bacteria typically regulate oxygen levels in the intestinal microenvironment; their loss may create anaerobic niches, indirectly impairing nutrient metabolism [5,55,57]. Tetracycline had no statistically significant effects on microbial functions, consistent with its narrower fitness impact (limited to fecundity), and its depletion of Actinomycetota (with limited functional redundancy in the TC group) may have targeted only specific reproductive symbionts without broadly disrupting metabolic pathways [11,39].

### 4.4. Advantages of Microbial Regulation-Based Pest Control Strategies and Study Limitations

The regulatory mechanisms of antibiotics on the microbiome and fitness of *H. cunea* revealed in this study provide a reference for green pest control strategies based on microbiome manipulation. Compared with traditional chemical pesticide control, such microbial regulation strategies exert control effects by interfering with the interaction network between pests and their essential symbiotic microorganisms, which can significantly reduce toxic damage to non-target organisms such as pollinators and beneficial soil microorganisms while avoiding issues of chemical pesticide residues [58,59]. Unlike traditional biological control that relies on natural enemy insects or pathogenic microbial agents, they can also more easily achieve stable control effects by precisely targeting the core functional gut microbiota of pests [2,15]. It should be clearly noted, however, that antibiotics used in this study only served a tool for mechanism exploration and cannot be directly applied in the field, as excessive antibiotic residues can induce the production of resistance genes that can transfer through the food chain [47,53] and threaten ecological and public health security. Thus, future efforts need to shift toward non-antibiotic approaches such as specific metabolic inhibitors and probiotic preparations to retain the targeting and stability advantages of microbial regulation while completely avoiding the ecological and health risks caused by antibiotic resistance.

This study clarified the differential regulatory mechanisms of three antibiotics on the microbiome and fitness of *H. cunea*, but there are still three limitations that can be addressed in subsequent research. First, only a single concentration of 0.5% (*w*/*v*) was used for antibiotic treatment. Although this concentration was selected with reference to similar insect microbiome studies [25,26,43] and had ecological simulation value, it cannot reveal the dose–effect relationship of different antibiotic concentrations on the larval microbiome and fitness, which may limit the analysis of the “antibiotic–microbe–host” interaction mechanism. Second, the antibiotic residue content in mulberry leaves after treatment was not quantified. Although the process of “soaking at 4 °C in the dark for 24 h + removing surface liquid with sterile filter paper + air-drying at room temperature” ensured the consistency of comparison between groups, differences in the physicochemical properties of different antibiotics could lead to variations in their adsorption efficiency and penetration depth in mulberry leaves, making it impossible to accurately quantify the actual antibiotic dose ingested by larvae. Third, this study confirmed the correlation between microbiome changes and larval fitness outcomes, but had not established a causal relationship between the two. The observed fitness changes may arise from two potential pathways: one was the direct toxic effects of antibiotics on *H. cunea* larvae (e.g., disrupting host cellular metabolism), and the other was the indirect effects mediated by microbiome disturbance (e.g., loss of symbiotic bacteria involved in amino acid synthesis).

In the future, multiple concentration gradients could be set and combined with dose–effect models to clarify the response thresholds of the microbiome and fitness to concentrations. Meanwhile, high-performance liquid chromatography (HPLC) could be used to detect antibiotic residues in mulberry leaves, and the actual exposure dose could be calculated by combining larval daily food intake. Additionally, conduct microbiome reconstitution experiments to verify whether restoring specific microbial taxa (e.g., Bacillota, Actinomycetota) can reverse the fitness decline trend of larvae in the antibiotic-treated groups, thereby establishing the causal relationship between microbiome disturbance and reduced host fitness. This will improve the rigor of the conclusions and lay a foundation for the ecological risk assessment of antibiotics on non-target insects and the development of green pest control strategies based on microbial regulation.

## 5. Conclusions

This study demonstrates that tetracycline, rifampicin, and kanamycin disrupt the microbiome and fitness of *H. cunea* through distinct mechanisms, arising from their intrinsic antibiotic specificity and differential effects on microbial diversity, structure, and function. These findings advance our understanding of antibiotic-mediated host–microbe interactions and provide a theoretical basis for ecological risk assessment of antibiotics on non-target insects and microbe-based green pest management.

## Figures and Tables

**Table 1:** Sequencing profile and alpha diversity indices of bacterial microbiota in *H. cunea*.

Sample Types	Read Counts	Observed Species Number	ACE Richness Index	Chao1 Richness Index	Shannon Diversity Index	Simpson Diversity Index	Sequencing Coverage
CK	64,469 ± 3551.232	124.333 ± 30.139	128.15 ± 28.831	127.503 ± 28.071	1.62 ± 0.11	0.367 ± 0.074	0.9998 ± 0
TC	52,413.333 ± 14,571.579	85.333 ± 9.504	86.572 ± 10.323	86.083 ± 9.919	1.526 ± 0.249	0.395 ± 0.116	0.9999 ± 0.0001
RFP	89,808.333 ± 23,278.021	97 ± 37.269	100.173 ± 36.076	99.95 ± 35.231	1.877 ± 1.163	0.399 ± 0.33	0.9999 ± 0
KAN	64,490 ± 61.879	84.667 ± 21.595	92.714 ± 22.642	91.291 ± 23.223	0.982 ± 0.274	0.625 ± 0.099	0.9997 ± 0

Data are presented as mean ± standard deviation (SD). Sample abbreviations are as in Figure 1.

## Data Availability

The *16S rRNA* sequencing data have been submitted and stored in the National Center for Biotechnology Information (NCBI) Sequence Read Archive (SRA) [https://www.ncbi.nlm.nih.gov/sra] with the BioProject accession number PRJNA1313420.

## Supplementary Materials

The following supporting information can be downloaded at https://www.mdpi.com/article/10.3390/microorganisms13092078/s1, Dataset S1: Standardized data for bacterial *16S rRNA* gene sequencing across four *H. cunea* strains.
